# Supplementary material for: TMS provokes target-dependent intracranial rhythms across human cortical and subcortical sites
Source: Brain Stimul. Author manuscript; Available in PMC 2024 Aug 9. (PMC11313454; doi:10.1016/j.brs.2024.05.014)
Supplement: 1 [file NIHMS2004001-supplement-1.docx]

Supplementary Materials for

**TMS provokes target-dependent intracranial rhythms across human cortical and subcortical sites**

Ethan A. Solomon* *et al.*

*Corresponding author. Email: [esolom@stanford.edu](mailto:esolom@stanford.edu)

**This file includes:**

Figs. S1 to S8

Tables S1


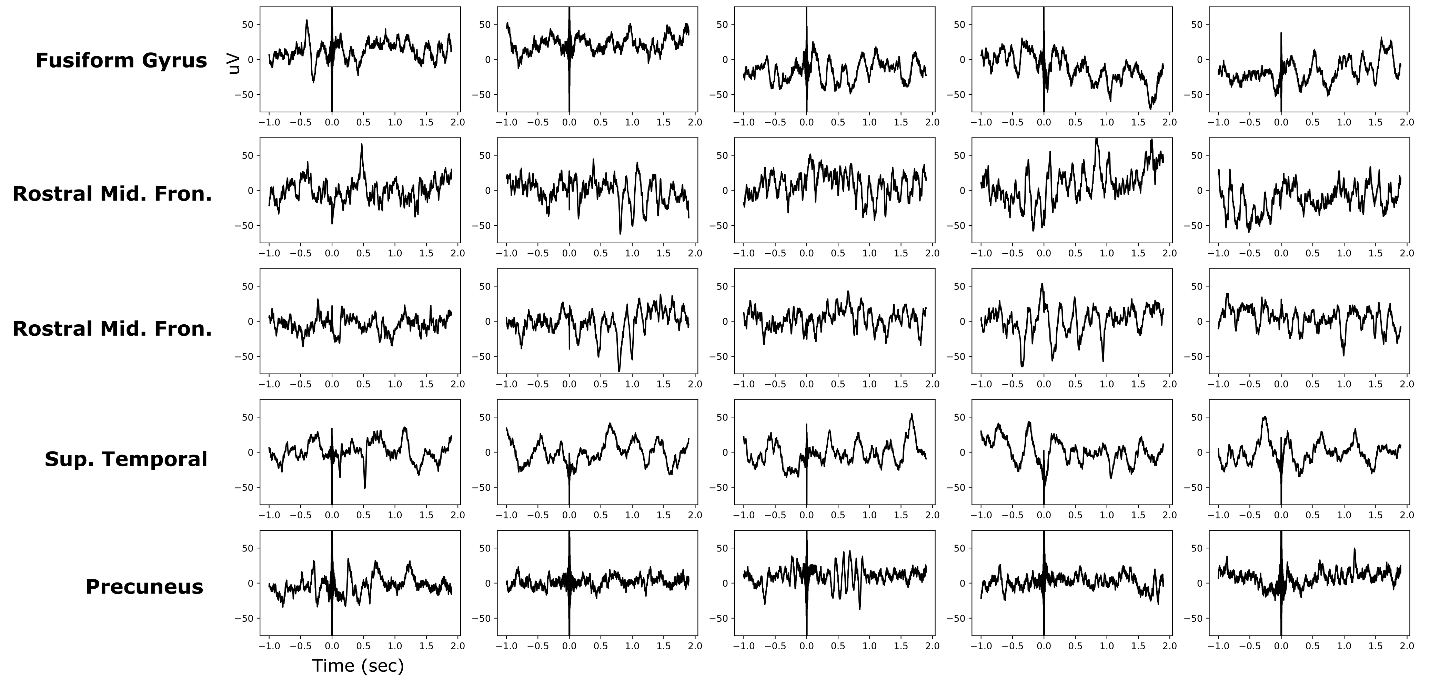


**Figure S1. Example raw EEG traces from 5 recording channels in a single subject, prior to artifact removal.** Electrode regions are indicated on the left. Each column corresponds to a randomly-selected trial of active TMS.

**
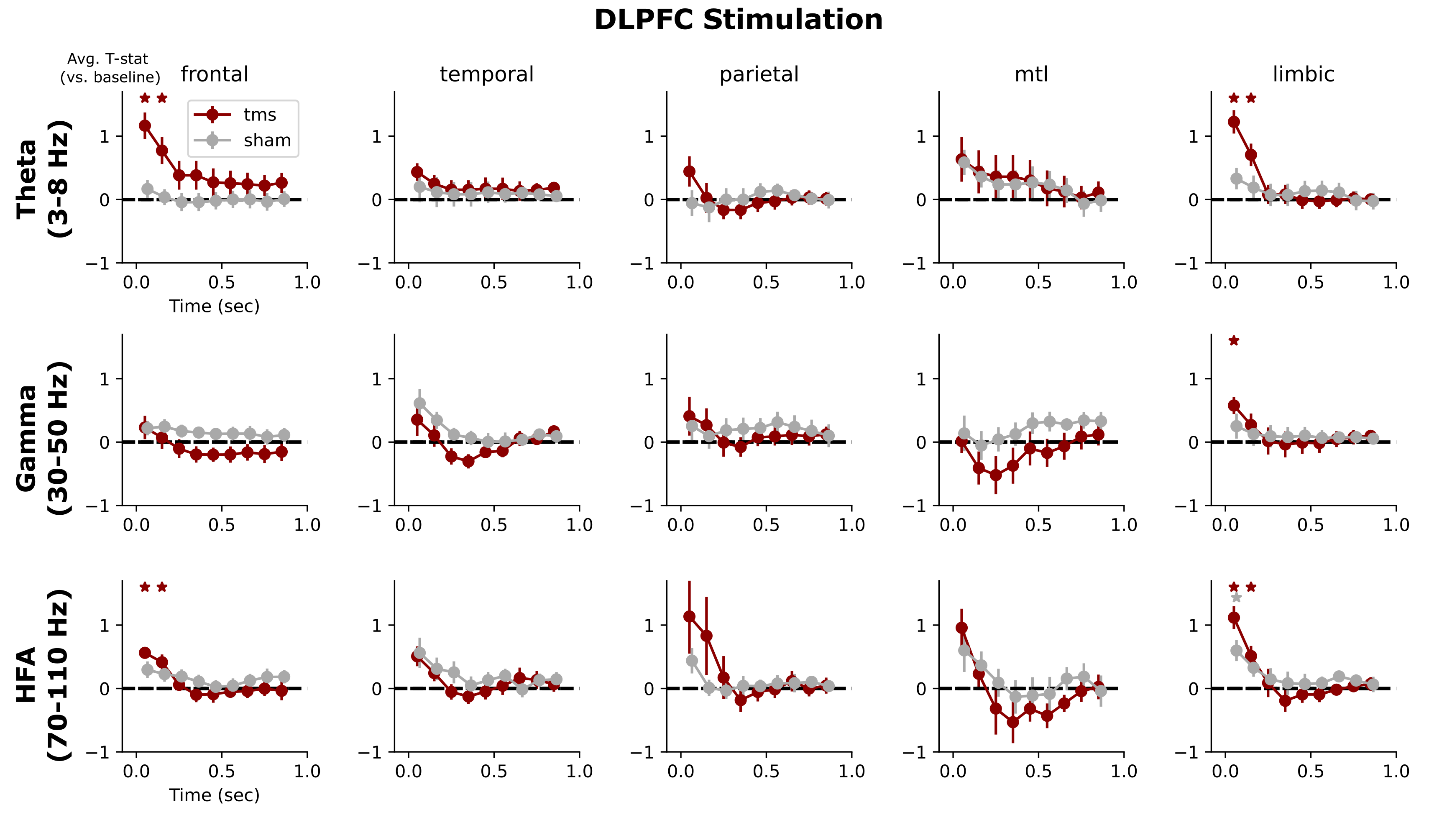
**

**
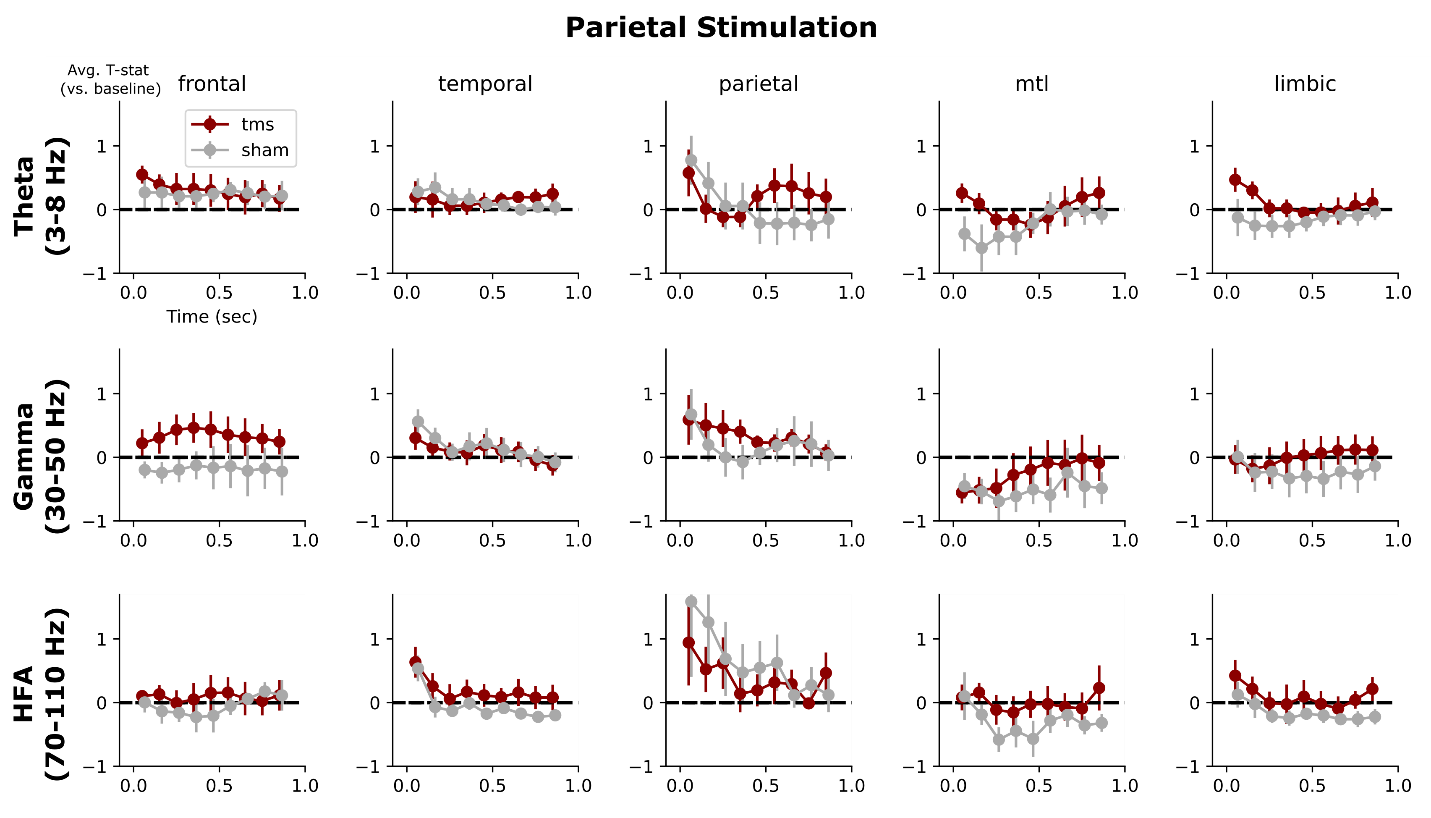
**

**Figure S2. Spectral responses within active and sham conditions relative to pre-stimulation baseline.** To assess for significant spectral effects within active TMS (red) or sham (gray) conditions alone – as opposed to the contrast between them – baseline spectral power (-500ms to -50ms) was subtracted from post-stimulation power for each electrode in the dataset, and then assessed for significance via 1-sample *t-*tests across subjects. Effects were aggregated in the same lobe-level regions-of-interest as presented in Figure 2. **p*<0.05, FDR corrected across timepoints. X-axis labels indicate the start point of 500ms (theta, gamma) or 250ms (HFA) windows.

**
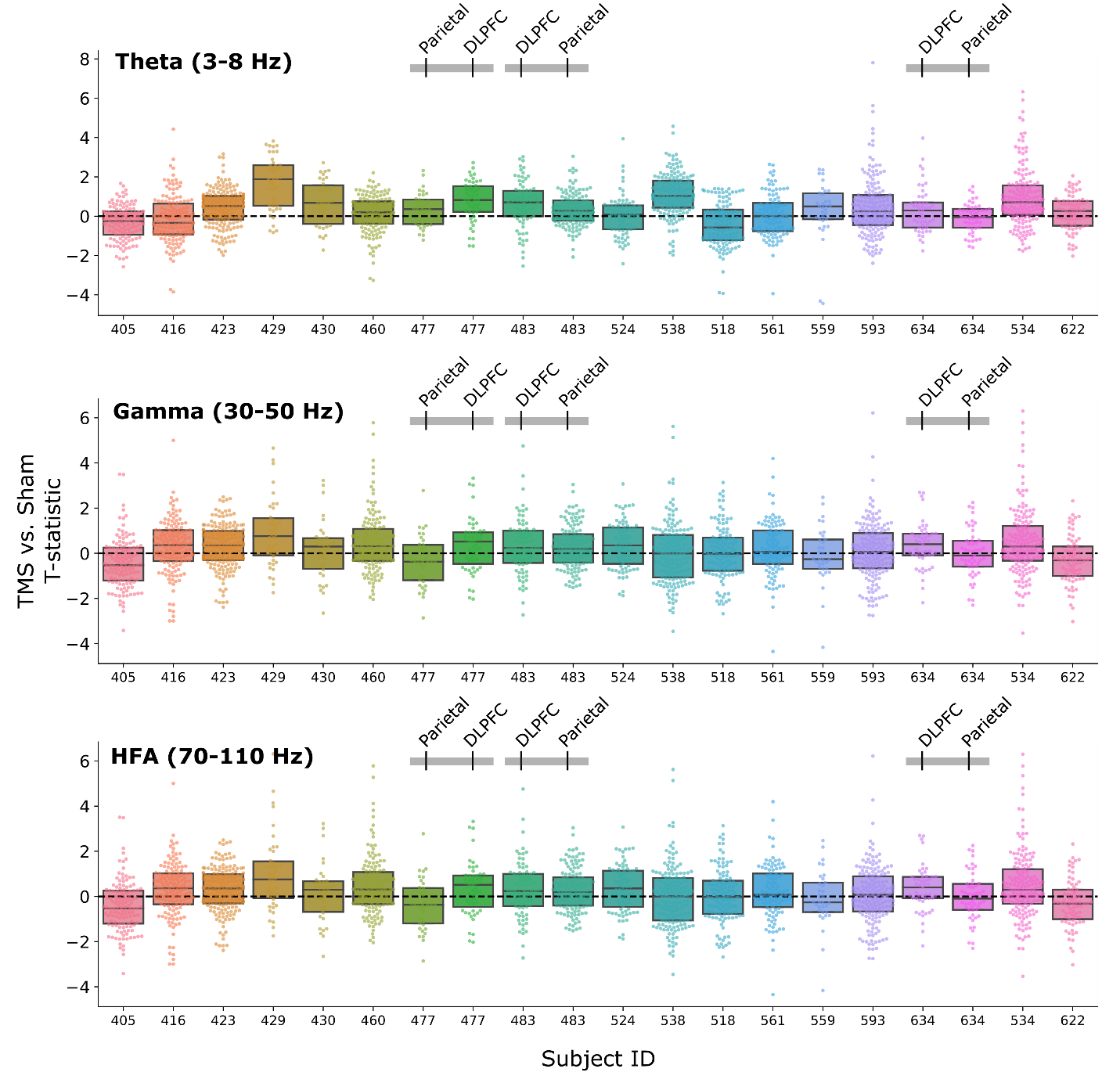
**

**Figure S3. TMS elicits brain-wide modulations of spectral power.** For each recording contact in the dataset, a *t*-statistic is computed which reflects the TMS-related change in spectral power relative to sham in the 50-500ms (theta, gamma) or 50-250ms (HFA) interval (see *Methods* and **Figure 1** for details). The distribution of *t*-statistics across all subjects and contacts is shown for each frequency band of interest (theta, gamma, and HFA). Three subjects (477, 483, 634) underwent stimulation at both parietal and DLPFC targets, indicated in the key above each plot. Boxes represent median and interquartile range.


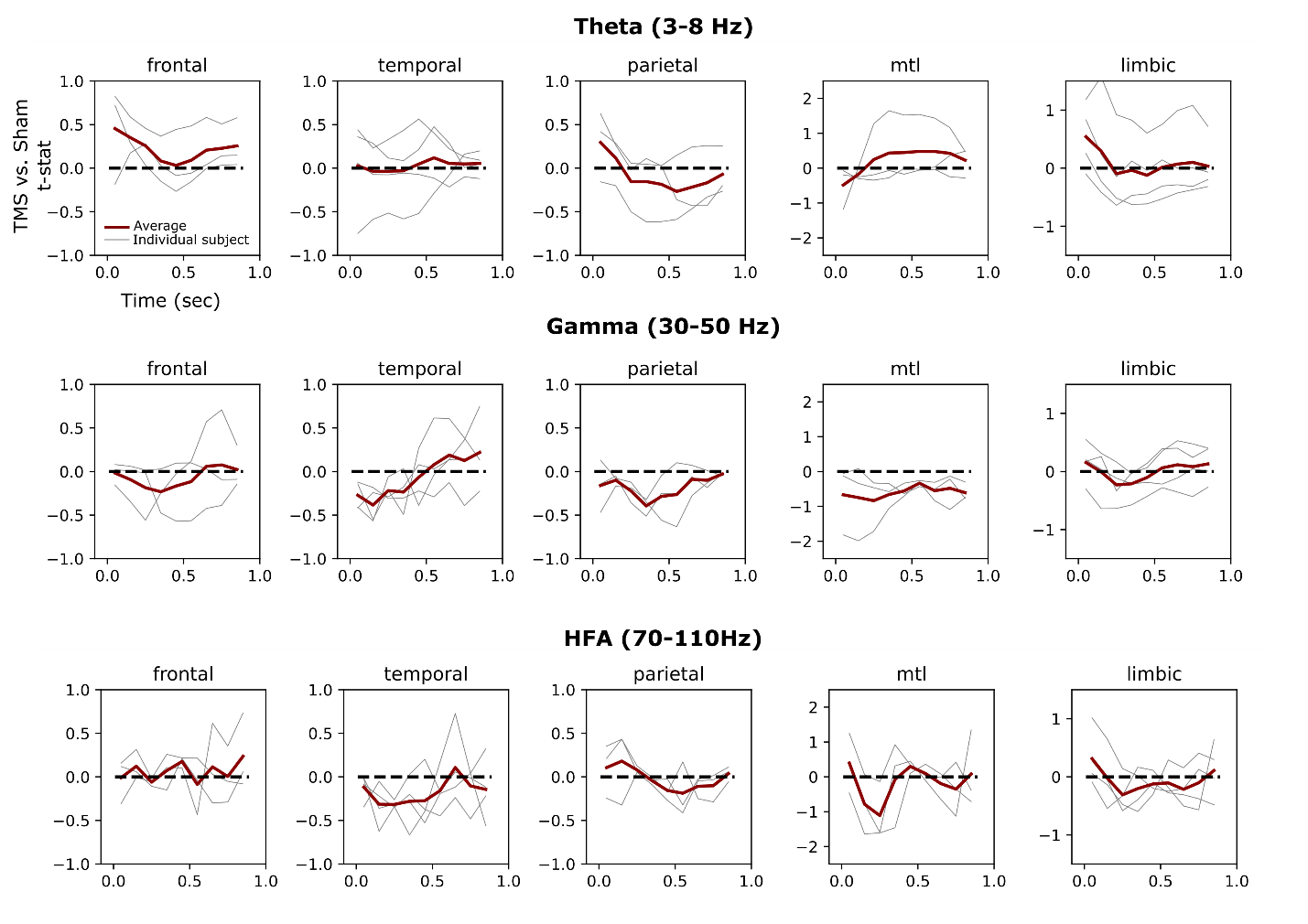


**Figure S4. DLPFC TMS-vs.-sham spectral power in the experimental sessions using cutaneous electrical stimulation during sham trials (N=4).** By delivering cutaneous electrical stimulation at the site of coil placement during sham trials, we aimed to better approximate the somatosensory responses from active TMS. Data were otherwise analyzed exactly as in Figure 2A. The small number of subjects precludes statistically robust conclusions, though it can be qualitatively seen that this subset replicates the main findings of early increases in frontolimbic theta as well as temporal decreases in gamma and HFA. Cutaneous stimulation was not paired with parietal stimulation in this dataset. X-axis labels indicate the start point of 500ms (theta, gamma) or 250ms (HFA) windows.

**
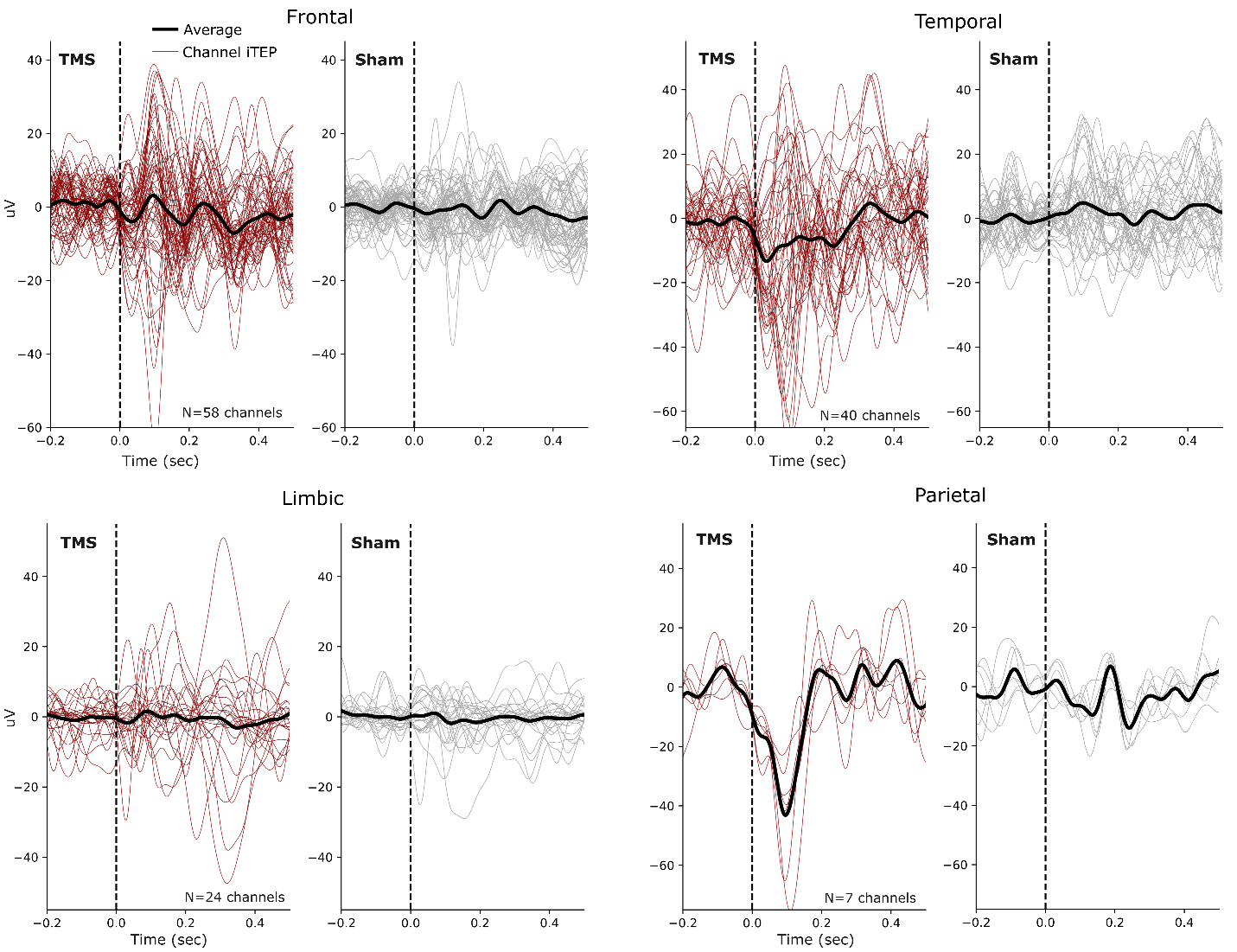
**

**Figure S5. Representative example iTEPs from key lobe-level ROIs following DLPFC stimulation.** Example intracranial DLPFC-targeted TMS-evoked potentials (iTEPs) are shown for the four major lobe-level ROIs used in our dataset. Individual traces correspond to channel-level iTEPs, while black lines shown channel-averaged iTEPs within a given region. Note that, due to heterogenous sampling across subjects, each lobe-level example derives from a different subject.


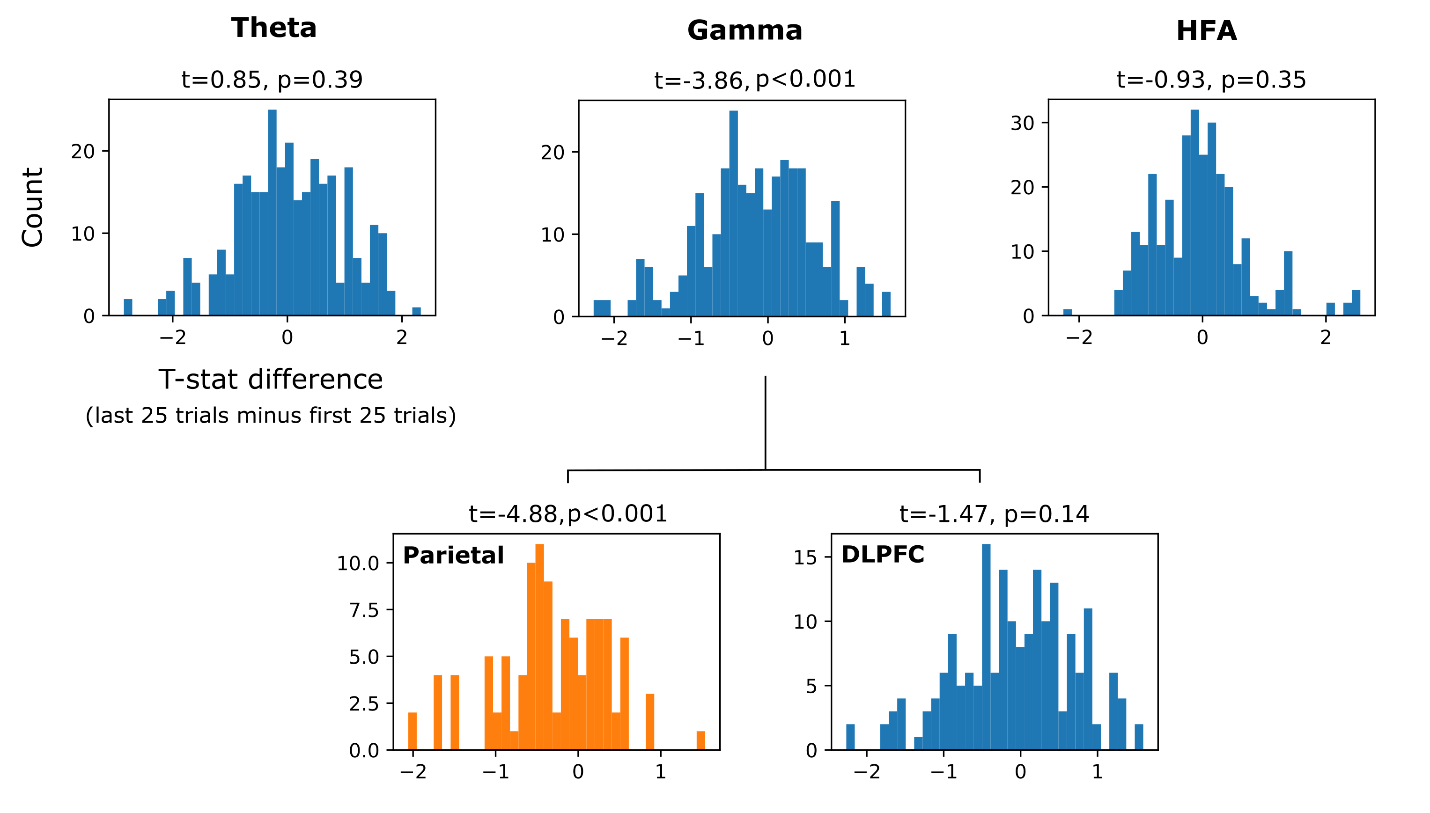


**Figure S6. Assessment of changes in TMS-related spectral power from beginning to end of stimulation sessions.** To understand whether 0.5Hz stimulation results in differential spectral effects over the length of a stimulation session, we separately reanalyzed the first 25 trials and last 25 trials of each stimulation session, recomputing *t*-statistics for each lobe-level ROI within each subject (see *Methods* for details). In this analysis, we included stimulation delivered to either the DLPFC or inferior parietal cortex. We then found the pairwise difference between these *t*-statistics to understand whether spectral power significantly differs between the early and later trials in a stimulation session. In the theta and HFA bands, there was no significant difference in TMS-related spectral power between the early and late periods of a stimulation session (1-sample *t-*test, *p*>0.05). In the gamma band, there was a significant decrease in TMS-related spectral power across the length of a session (*t*=-3.86, *p*<0.01), indicating that stimulation tended to cause greater decreases in gamma power near the end of a stimulation session. Individually testing trials of parietal and DLPFC stimulation, we found a significant effect for parietal stimulation (*t*=-4.88, *p*<0.001).

**
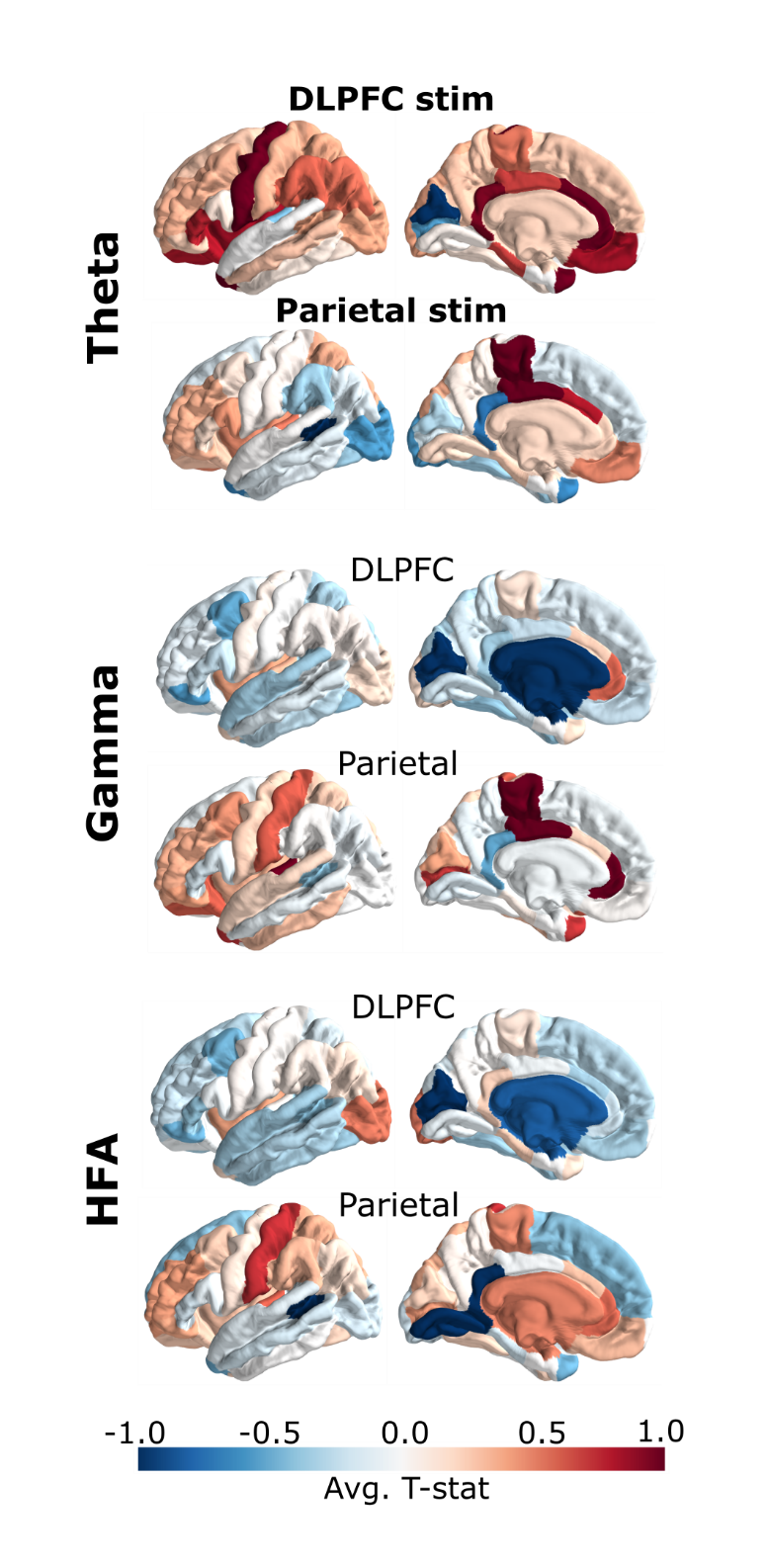
**

**Figure S7. Average TMS vs. Sham evoked power across DKT regions in complete dataset.** Exactly as **Figure** **3D**, but without a threshold for minimum number of subjects per ROI.

**
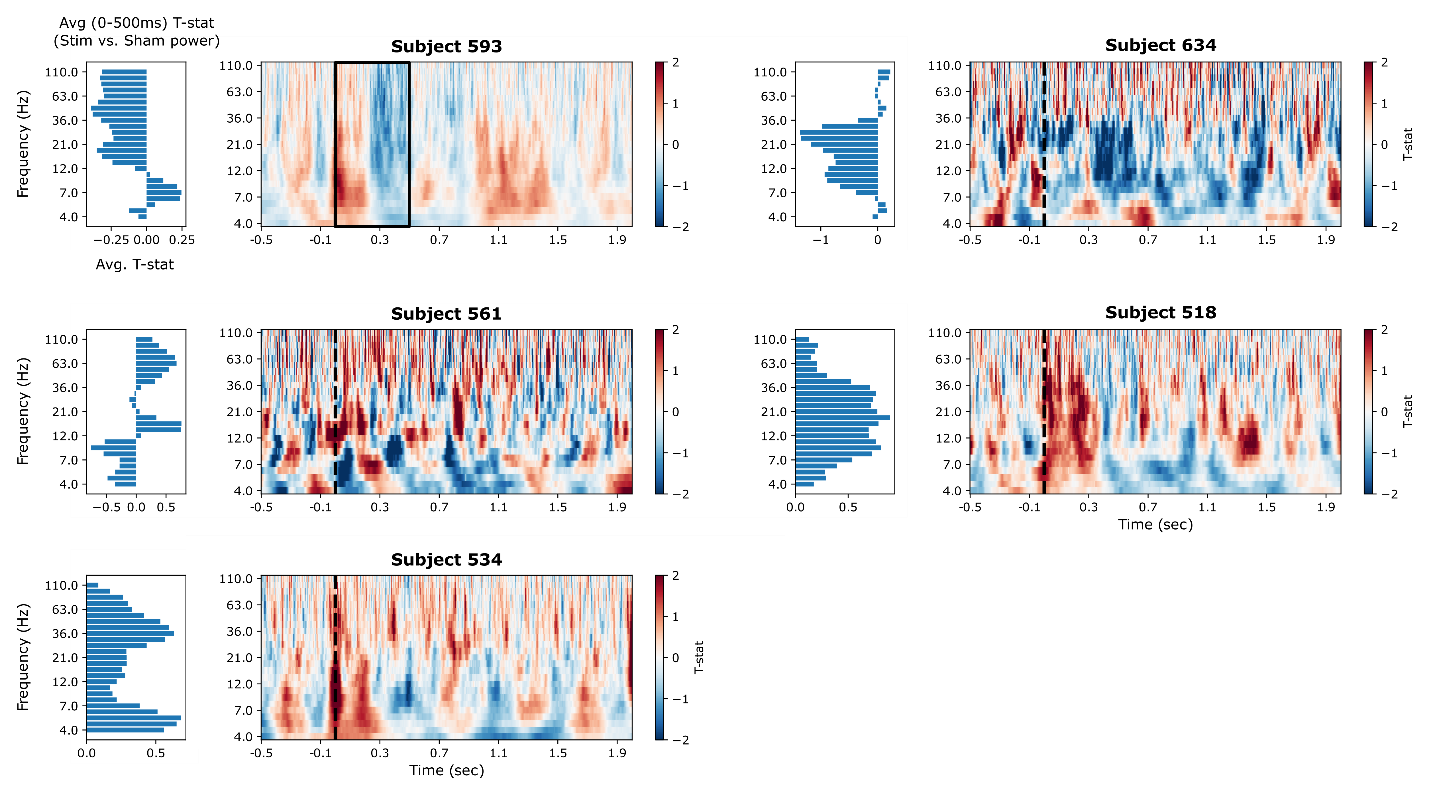
**

**Figure S8. Amygdala spectral power across individual subjects following DLPFC stimulation.** Time-frequency representations for each of the 5 subjects who had recording contacts in the amygdala and underwent TMS to the DLPFC. Time-frequency responses are constructed as described in **Figure 3A** and *Methods*. To the left of each time-frequency plot is the average power at each frequency within the 0-500ms post-stimulation interval. Subjects demonstrate heterogeneous effects with differing peak power for each subject; none show an entirely broadband response that would suggest contamination by stimulation artifact.

**Table S1. DKT regions included in broad ROIs.**

| **ROI** | **Constituent DKT labels** |
| --- | --- |
| Frontal | lateralorbitofrontal, parsorbitalis, precentral, caudalmiddlefrontal, parsopercularis, paracentral, rostralmiddlefrontal, medialorbitofrontal, superiorfrontal, parstriangularis, |
| Temporal | inferiortemporal, fusiform, temporalpole, superiortemporal, middletemporal, transversetemporal, bankssts |
| Parietal | supramarginal, inferiorparietal, superiorparietal, precuneus, cuneus |
| MTL | entorhinal, parahippocampal, Hippocampus |
| Limbic | entorhinal, insula, isthmuscingulate, posteriorcingulate, Amygdala, caudalanteriorcingulate, rostralanteriorcingulate, parahippocampal, Hippocampus |
